# Comparison of the metabolic activation of environmental carcinogens in mouse embryonic stem cells and mouse embryonic fibroblasts

**DOI:** 10.1016/j.tiv.2014.09.004

**Published:** 2015-02

**Authors:** Annette M. Krais, Karl-Rudolf Mühlbauer, Jill E. Kucab, Helena Chinbuah, Michael G. Cornelius, Quan-Xiang Wei, Monica Hollstein, David H. Phillips, Volker M. Arlt, Heinz H. Schmeiser

**Affiliations:** a Analytical and Environmental Sciences Division, MRC-PHE Centre for Environment and Health, King’s College London, London, United Kingdom; b Research Group Genetic Alterations in Carcinogenesis, German Cancer Research Center (DKFZ), Heidelberg, Germany; c Molecular Mechanisms and Biomarkers Group, International Agency for Research on Cancer, Lyon, France

**Keywords:** Mouse embryonic stem cells, Mouse embryonic fibroblasts, Carcinogen activation, Cytochrome P450, NADP(H)quinone oxidoreductase, DNA adduct formation

## Abstract

We compared mouse embryonic stem (ES) cells and fibroblasts (MEFs) for their ability to metabolically activate the environmental carcinogens benzo[*a*]pyrene (BaP), 3-nitrobenzanthrone (3-NBA) and aristolochic acid I (AAI), measuring DNA adduct formation by ^32^P-postlabelling and expression of xenobiotic-metabolism genes by quantitative real-time PCR. At 2 μM, BaP induced *Cyp1a1* expression in MEFs to a much greater extent than in ES cells and formed 45 times more adducts. *Nqo1* mRNA expression was increased by 3-NBA in both cell types but induction was higher in MEFs, as was adduct formation. For AAI, DNA binding was over 450 times higher in MEFs than in ES cells, although *Nqo1* and *Cyp1a1* transcriptional levels did not explain this difference. We found higher global methylation of DNA in ES cells than in MEFs, which suggests higher chromatin density and lower accessibility of the DNA to DNA damaging agents in ES cells. However, AAI treatment did not alter DNA methylation. Thus mouse ES cells and MEFs have the metabolic competence to activate a number of environmental carcinogens, but MEFs have lower global DNA methylation and higher metabolic capacity than mouse ES cells.

## Highlights

• ES cells and MEF have the metabolic competence to activate environmental carcinogens.
• Carcinogen-induced genotoxicity in MEFs is higher than in ES cells.
• MEFs have higher metabolic capacity than ES cells.
• Metabolic capacity depends on the carcinogen studied.

## 1 Introduction

The protein p53, encoded by *TP53*, is a transcription factor that induces cell cycle arrest, apoptosis and DNA repair in response to cellular stress and DNA damage in order to protect the cell from oncogenic transformation, which has led to its description as ‘the guardian of the genome’ (Lane, 1992). Disruption of the normal p53 response by *TP53* mutation leads to the development of tumours and as 50% of human tumours contain a mutation in *TP53* it is arguably the most important cancer gene (Olivier et al., 2010).

Mouse models offer the possibility to study p53 function both through phenotypic analysis of the whole organism and through examination of a variety of primary cell types derived from mice (Kenzelmann Broz and Attardi, 2010). These models include knockout of *Trp53* to study loss of p53 function and knock-in strategies to examine human *TP53* mutants and polymorphic variants. For example, studies in mouse strains expressing mutant p53 corresponding to R175H and R273H hot spot mutations in human cancers revealed that these mutants exhibited gain-of-function properties in addition to loss of normal p53 function (*i.e.* altered tumour spectrum in addition to more metastatic tumours) (Freed-Pastor and Prives, 2012, Lang et al., 2004, Olive et al., 2004). In another study Song et al. (2007) introduced two common human *TP53* cancer mutations, R248W and R273H, independently into humanized *TP53* knock-in (Hupki) mice and found that the tumour suppressor functions of p53 were abolished in mice with mutant p53. Further, their findings suggested that mutant, but not wild-type, p53 can interact with and inhibit ATM, a protein involved in the recognition of DNA damage, indicating that p53 gain-of-function mutants can promote tumourigenesis by interfering with critical DNA damage response pathways (Song et al., 2007).

We have used the Hupki model to study carcinogen-induced *TP53* mutagenesis where primary Hupki embryo fibroblasts (HUFs) were exposed to mutagens and then selected for bypass of culture-induced senescence and immortalisation (Kucab et al., 2010, Luo et al., 2001). Environmental carcinogens that have been examined using the HUF immortalisation assay include benzo[*a*]pyrene (BaP), which is associated with tobacco smoke-induced lung cancer (Liu et al., 2005, Reinbold et al., 2008) and aristolochic acid (AA), which is linked to aristolochic acid nephropathy (AAN)-associated urothelial cancer (Gokmen et al., 2013, Liu et al., 2004, Nedelko et al., 2009). In both cases the generated *TP53* mutation pattern corresponded to the pattern found in human tumours (Hollstein et al., 2013, Kucab et al., 2010).

The p53 Platform (PLF) mouse is a novel mouse strain which allows the precise importation of human *TP53* sequences into the endogenous mouse *Trp53* gene (Wei et al., 2011, Wei et al., 2012). Integrase-mediated cassette exchange in PLF embryonic stem (ES) cells or mouse embryonic fibroblasts (MEFs) is an efficient way to generate kindred of distinct mutant clones that are closely matched in genetic background for comparative functional analysis of p53 (Wei et al., 2012). The system not only allows one to determine the extent to which a mutation compromises p53 wild-type function (Odell et al., 2013) but may also provide a powerful tool to study the response of cells carrying mutant p53 to cellular stress and DNA damage. Recent findings have indicated that wild-type p53 can impact on the bioactivation of environmental carcinogen and drugs indicating that the cellular *TP53* status is linked to the regulation of xenobiotic-metabolising enzymes (XMEs) (Goldstein et al., 2013, Hockley et al., 2008, Simoes et al., 2008). Thus as mutant p53 expressed in preneoplastic and/or neoplastic cells severely limits or abolishes the capacity of p53 to regulate its target genes (Freed-Pastor and Prives, 2012), mutant p53 may also impact on the expression of XMEs.

Prior to studying carcinogen-induced cellular responses of p53 mutated ES cells and MEFs derived from the PLF mouse it must be ensured that they are metabolically competent to activate the carcinogen studied. We showed previously that primary HUFs have the metabolic capacity to activate some environmental carcinogens including BaP, AAI and the air pollutant 3-nitrobenzanthrone (3-NBA), all of which have also been studied in the HUF immortalisation assay and are capable of inducing *TP53* mutations (Liu et al., 2004, Liu et al., 2005, Nedelko et al., 2009, Reinbold et al., 2008, Brocke et al., 2009). However, little is known about the metabolic competence of mouse ES cells with regard to environmental carcinogens. In the present study we have compared ES cells and MEFs derived from mice on a C57Bl/6 background, the same genetic background as the PLF mouse, for their ability to metabolically activate the carcinogens BaP, 3-NBA and AAI. Thus, these results are important for future studies using ES cells and MEFs derived from the PLF mouse carrying mutant p53. DNA adduct formation was assessed by ^32^P-postlabelling and the DNA damage response proteins p53 and p21 were evaluated by Western blotting. We also determined by quantitative real-time PCR (qRT-PCR) the gene expression of two selected enzymes, cytochrome P450 1a1 (Cyp1a1) and NADP(H)quinone oxidoreductase (Nqo1).

## 2 Material and methods

### 2.1 Carcinogens

Benzo[*a*]pyrene (BaP) and aristolochic acid I (AAI, as sodium salt) were obtained from Sigma Aldrich (Gillingham, UK). 3-Nitrobenzanthrone (3-NBA) was synthesised as described (Arlt et al., 2002).

### 2.2 Mouse breeding and isolation of murine embryonic stem cells (ES) and murine embryonic fibroblasts (MEFs)

In the PLF mouse, exons 2-9 of the mouse *Trp53* gene have been replaced by a PGK-neomycin resistance gene cassette to allow efficient exchange of the PGK-neo cassette with an incoming human *TP53* sequence of interest (Wei et al., 2011, Wei et al., 2012). The modified *Trp53* allele is the designated platform (*plf*) allele, where the *plf*/*plf* genotype is nominally p53 null and *plf*/*Trp53* retains one functional mouse *Trp53* allele along with the *plf* allele. Heterozygous p53 PLF mice (*plf*/*Trp53*; on a C57Bl/6 background) were bred at the Animal Facility of the German Cancer Research Center and were kept under standard conditions with food and water ad libitum. This breeding strategy allows for the generation of progeny with the same genetic background but differing in *Trp53* locus. Sibling embryos can be harvested with or without the *plf* allele. The reason for this breeding scheme is that a homozygous *plf* colony is difficult to maintain due to the short life expectancy of *plf*/*plf* (p53 null) mice. Sibling embryos that are *Trp53*/*Trp53* (*i.e.* with no *plf* allele) are not PLF mice and thus representative of a normal wild-type p53 laboratory mouse strain but have the same genetic background (*i.e.* C57Bl/6) as PLF mice. All animal procedures were carried out under licence in accordance with the law, and with local ethical review.

Isolation of mouse ES cells was performed as described previously (Wei et al., 2011). Briefly, 2.5 day-old morulas were isolated, denuded and plated on a feeder layer (Tesar, 2005). Three days after plating, attached structures were isolated, trypsinised and reseeded until clones with appropriate morphology were harvested (Wei et al., 2011). The ES cells used in this study were from the F2 clone (*Trp53*/*Trp53*) which have wild-type p53.

To obtain primary embryonic fibroblasts, day 13.5 *Trp53*/*Trp53* embryos were harvested according to a standard protocol, and fibroblasts were isolated from each embryo as described previously (Liu et al., 2007). Briefly, neural and hematopoietic tissue was removed from each embryo by dissection. The remaining tissue was minced and then trypsinised at 37 °C for 5 min. Cells were grown under standard conditions (see below) to 100% confluence before preparing frozen stocks (passage 0). These MEFs on a C57Bl/6 background have wild-type p53.

### 2.3 Cell culture and carcinogen treatment

Mouse ES cells were cultured at 37 °C and 5% CO_2_ in Dulbecco’s modified Eagle’s medium (DMEM), high glucose (4.5 g/L), supplemented with 15% of ES Cell Fetal Bovine Serum (FBS; PAN Biotech, Aidenbach, Germany), 2 mM l-glutamine, 1 × MEM non-essential amino acids (11140050; Invitrogen, Darmstadt, Germany), 1 mM sodium pyruvate, 100 U/mL antibiotics (15140122; Gibco; penicillin and streptomycin), 100 μM of 2-mercaptoethanol (Sigma, Taufkirchen, Germany) and 1000 U/mL leukemia inhibitory factor (LIF) ESGRO (Millipore, Darmstadt, Germany). Cell culture dishes used for ES cells were pre-coated with 0.2% gelatin (dissolved in PBS, Invitrogen, Germany) at room temperature for at least one hour which was removed just prior to use. MEFs were cultured at 37 °C and 5% CO_2_ in DMEM, high glucose (4.5 g/L) supplemented with 10% FBS (PAN), 2 mM l-glutamine, 1 mM sodium pyruvate and 100 U/mL antibiotics (penicillin and streptomycin). All cell culture reagents were purchased from Invitrogen (Germany) unless stated otherwise.

Cells were seeded 48 h prior to carcinogen treatment with BaP, 3-NBA and AAI. BaP and 3-NBA were dissolved in dimethyl sulfoxide (DMSO); the DMSO concentration was always kept at 0.5% of the total culture medium volume. AAI was dissolved in water. Cells treated with solvent only were used as controls.

### 2.4 Cell viability and DNA adduct analysis

Cell numbers were counted using the Countess® Automated Cell Counter (Life Technologies, Darmstadt, Germany) and are represented as percentage of the control cell number.

DNA was isolated from carcinogen-treated cells using standard phenol/chloroform extraction method. DNA adduct formation was analysed by ^32^P-postlabelling as described with minor modifications (Schmeiser et al., 2013). Briefly, 6.25 μg DNA were digested using micrococcal endonuclease (375 mU/sample; Sigma, Taufkirchen, Germany) and spleen phosphodiesterase (31.25 mU/sample; Worthington, Lakewood, NJ, USA) for 3 h at 37 °C. An aliquot (1.25 μg) of the digest was removed and diluted for determination of normal nucleotides. For BaP and AAI, adducts were enriched using nuclease P1 digestion, whereas for 3-NBA, adducts were enriched using butanol extraction as reported (Schmeiser et al., 2013). Subsequently, adducts were labelled by incubation with [γ-^32^P]ATP (50 μCi/sample; Hartmann-Analytic, Braunschweig, Germany) and T4-polynucleotide kinase (USB, Germany) for 30 min at room temperature.

^32^P-labelled adduct nucleoside bisphosphates were separated by thin-layer chromatography (TLC) on polyethylenimine (PEI)-cellulose sheets (Macherey-Nagel, Düren, Germany). The following solvents were used (Schmeiser et al., 2013): for all experiments − D1, 1 M sodium phosphate, pH 6.5; D5, 1.7 M sodium phosphate, pH 6.0; for BaP − D3, 3.5 M lithium formate, 8.5 M urea, pH 3.5; D4, 0.8 M lithium chloride, 0.5 M Tris, 8.5 M urea, pH 8.0; for 3-NBA − D3, 4 M lithium formate, 7.0 M urea, pH 3.5; D4, 0.8 M lithium chloride, 0.5 M Tris, 8.5 M urea, pH 8.0; for AAI − D3, 3.5 M lithium formate, 8.5 M urea, pH 4.0; D4, 0.8 M lithium chloride, 0.5 M Tris, 8.5 M urea, pH 9.0. After chromatography, electronic autoradiography of TLC sheets was performed using a Packard Instant Imager (Dowers Grove, IL, USA). DNA adduct levels (RAL, relative adduct labelling) were calculated as counts per minute (cpm) adducts per cpm normal nucleotides and expressed as adducts per 10^8^ normal nucleotides (Schmeiser et al., 2013). No DNA adduct spots were observed in control (untreated) cells (data not shown).

### 2.5 Western blot analysis

After treatment cells were lysed with 62.5 mM Tris-HCl pH 6.8, 500 mM EDTA pH 8.0, 2% sodium dodecyl sulphate (SDS) and 10% glycerol supplemented with fresh protease inhibitors (78425; Thermo Scientific, Loughborough, UK). Lysates were sonicated to shear genomic DNA and protein concentration was determined using the Pierce™ BCA Protein Assay Kit (Thermo Scientific, UK). Lysates were separated on sodium-polyacrylamide gel electrophoresis (SDS-PAGE) using NuPage 4-12% gels (Life Technologies, Paisley, UK) and transferred to nitrocellulose membranes by electroblotting as previously reported (Hockley et al., 2006). Membranes were blocked with 3% non-fat dried milk in Tris-buffered saline (TBS) + Tween (0.1%) for 1 h at room temperature and incubated overnight with primary antibody diluted in blocking buffer. The following antibodies were used: anti-p53 (1C12, mouse mAb #2524, 1:5000; Cell Signalling, Hitchin, UK); anti-p21 (mouse mAb #556431, 1:2000; BD Bioscience, Oxford, UK); and GAPDH (mouse mAb #MAB374, 1:10,000; Millipore, Watford, Hertfordshire, UK). Membranes were washed and incubated with horseradish peroxidase-conjugated goat anti-mouse secondary antibody (CST 7074, 1:10,000; Cell Signalling, UK). Proteins were visualised using the enhanced chemiluminescent SuperSignal West Pico detection reagent according to the manufacturer’s instruction (#34080; Thermo Scientific, UK).

### 2.6 Gene expression analysis

Prior to assessing the expression of XMEs, carcinogen treatment conditions were optimised to ensure, where possible, that sufficient DNA damage was induced without significant adverse effects on cell viability in order to compare DNA adduct formation both in ES cells and MEFs (Fig. 2).

**Fig. 1:**
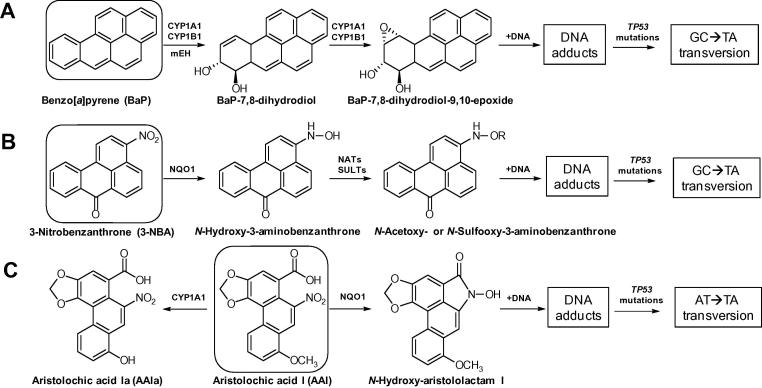
Metabolic activation and DNA adduct formation of (A) BaP, (B) 3-NBA and (C) AAI. R = –SO_3_H; R = –C(O)CH_3_.

**Fig. 2:**
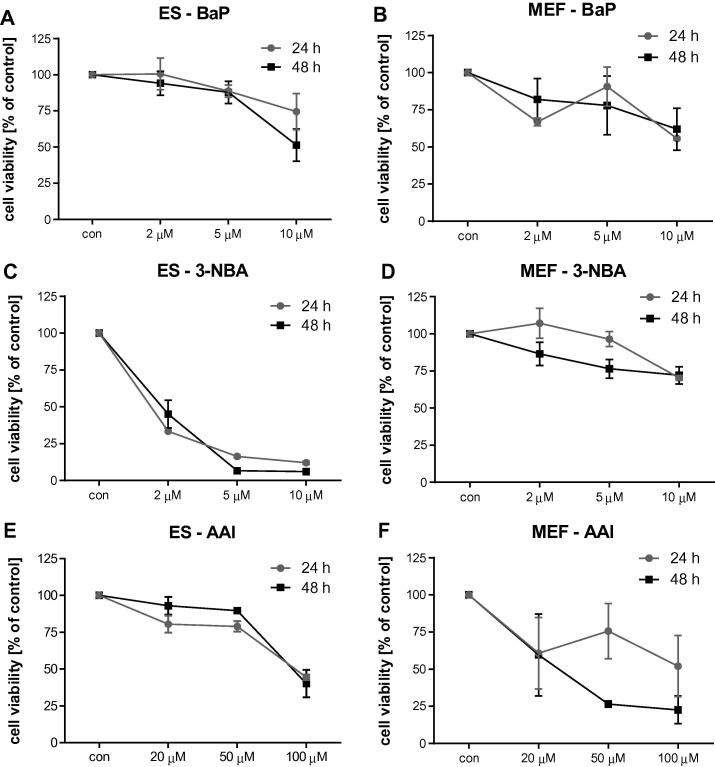
Effect of BaP (A and B), 3-NBA (C and D) and AAI (E and F) on cell viability (% control) of ES cells (*left panel*) and MEFs (*right panel*) derived from mice on a C57Bl/6 genetic background carrying wild-type *Trp53*. Values represent mean ± range of duplicate cell incubations.

Cells were washed in phosphate-buffered saline (PBS) and total RNA was extracted using the GenElute Mammalian Total RNA Miniprepkit (Sigma, UK). Reverse transcription was performed using random primers and SuperScript® III Reverse Transcriptase (Life Technologies, UK). RNA expression was analysed by quantitative real-time polymerase chain reaction (qRT-PCR) using TaqMan® Universal PCR Master Mix (Life Technologies) and TaqMan® gene expression primers according to the manufacturer’s protocol with a 7500HT Fast Real Time PCR System (Applied Biosystems, UK). Probes (Life Technologies, UK) used were Mm01253561_m1 (*Cyp1a1*) and Mm00487218 (*Nqo1*) and expression levels were normalised to *Gapdh* (4352341E). Relative gene expression was calculated using the comparative threshold cycle (*C_T_*) method (Kucab et al., 2012).

### 2.7 Global methylation analysis

DNA (1 μg) was dissolved in water (7.5 μL) and incubated for 3 h at 37 °C with a mixture of 2.1 μL of micrococcal endonuclease (150 mU/μL, Sigma, Germany) and spleen phosphodiesterase (12.5 mU/μL, Worthington, USA) and 0.4 μL buffer (250 mM HEPES, 100 mM calcium chloride pH 6.0). Hydrolyzed dNps were derivatised with BODIPY FL EDA as described before (Krais et al., 2011). Briefly, to the DNA digests was added: 15 μL HEPES buffer (50 mM, pH 6.5), 15 μL 1-ethyl-3-(3′-*N*,*N*′-dimethyl-aminopropyl)-carbodiimide hydrochloride (EDC; Sigma, Germany; 1.8 M in 50 mM HEPES buffer, pH 6.5, Sigma) and 15 μL 4,4-difluoro-5,7-dimethyl-4-bora-3a,4a-diaza-s-indacene-3-propionyl ethylene diamine hydrochloride (BODIPY FL EDA; Invitrogen, Germany; 27 mM in 50 mM HEPES buffer, pH 6.5). Samples were incubated for 25 h at 25 °C in the dark.

After overnight incubation, 30 μL of the reaction mixture was diluted with 270 μL water and then 300 μL of a solution of sodium tetraphenylborate (Merck, Darmstadt, Germany; 52.5 mM in 1 mM sodium phosphate buffer, pH 6.0) was slowly added to precipitate the excess of BODIPY FL EDA and EDC. After mixing, 10 mL methylene chloride was added, followed by vortex mixing and centrifugation for 4 min at 3000 rpm. The aqueous phase was removed and directly analyzed by capillary electrophoresis coupled with laser-induced fluorescence (CE-LIF). Correction factors were determined as described previously (Krais et al., 2011).

CE-LIF analysis was performed on a PACE™ MDQ system with a Laser System Sapphire 488 CW (*λ*_em_ = 488 nm) from Coherent (Germany). Electrolyte and separation conditions were: 90 mM SDS in a solution of 90% (*v*/*v*) sodium phosphate buffer (18 mM, pH 9.0) and 10% (*v*/*v*) methanol as organic modifier; fused-silica capillary column, total length 59 cm; length to the detection window 48.5 cm; inner diameter 50 μm; injection 2.5 psis; temperature 20 °C; applied voltage 20 kV. Data were collected and analysed using 32 Karat software (version 5.0, Beckman Coulter). Time corrected individual peak areas were determined as described previously (Krais et al., 2011).

## 3 Results and discussion

Mouse ES cells are increasingly being used in mechanism-based genotoxicity testing (Hendriks et al., 2012, Pines et al., 2011). They provide an attractive system as they are untransformed, continuously proliferating cells that are proficient in the main DNA damage signalling pathways and cell cycle control systems and are genetically stable (Hendriks et al., 2013). As most environmental carcinogens require metabolism to exert their genotoxic activity we compared ES cells and MEFs derived from mice on a C57Bl/6 genetic background carrying wild-type *Trp53* for their ability to metabolically activate environmental carcinogens. We selected a variety of environmental carcinogens of different chemical classes where the metabolism is well studied and characterised. The cell culture test conditions were based on previous studies using these carcinogens in mammalian cells (Arlt et al., 2007, Hockley et al., 2008, Kucab et al., 2012, Simoes et al., 2008). We used carcinogen-DNA adduct formation as a surrogate measure of the relevant XME activity as all tested environmental carcinogens induce specific and structurally-identified DNA adducts which can be detected by the ^32^P-postlabelling assay (Schmeiser et al., 2013).

### 3.1 Metabolic activation and DNA damage induced by BaP in ES cells and MEFs

**Fig. 3:**
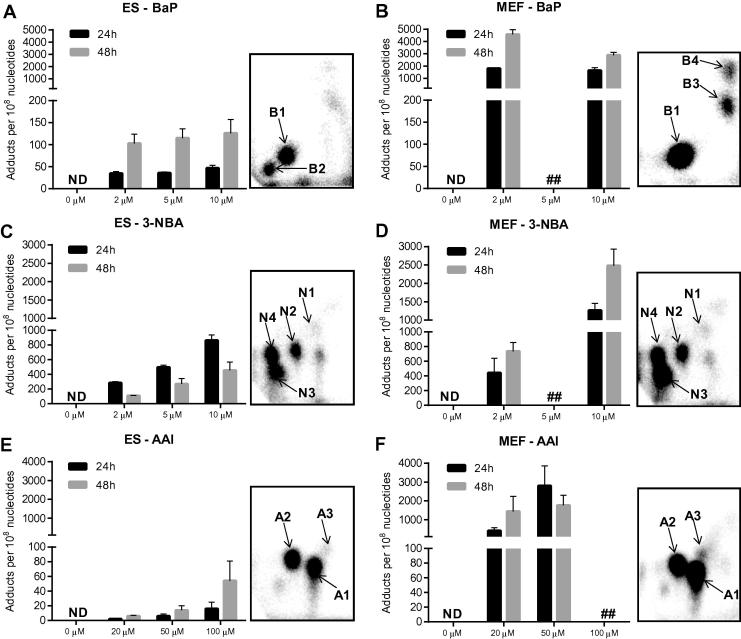
DNA adduct levels measured by ^32^P-postlabelling in ES cells (*left panel*) and MEFs (*right panel*) derived from mice on a C57Bl/6 genetic background carrying wild-type *Trp53* after exposure to BaP (A and B), 3-NBA (C and D) and AAI (E and F) for 24 and 48 h. Values are the mean ± SD of three incubations; each sample was determined by independent post-labelled analyses. Inserts: Autoradiographic profiles of DNA adducts formed in ES cells and MEFs after exposure; the origin, at the bottom left-hand corner, was cut off before exposure. B1, 10-(deoxyguanosin-*N*^2^-yl)-7,8,9-trihydroxy-7,8,9,10-tetrahydrobenzo[*a*]pyrene (dG-*N*^2^-BPDE); B2, probable guanine adduct derived from reaction with 9-hydroxy-BaP-4,5-epoxide; B3 and B4, uncharacterised BaP-derived DNA adducts; N1, 2′(2′-deoxyadenosine-*N*^6^-yl)-3-aminobenzanthrone (dA-*N*^6^-3-ABA); N2, as-yet unidentified adenine adduct derived from nitroreduction; N3, *N*-(2′-deoxyguanosine-*N*^2^-yl)-3-aminobenzanthrone (dG-*N*^2^-3-ABA); N4, *N*-(2′-deoxyguanosin-8-yl)-3-aminobenzanthrone (dG-C8-*N*-3-ABA); A1, 7-(deoxyadenosine-*N*^6^-yl)aristololactam I (dA-AAI); A2, 7-(deoxyguanosin-*N*^2^-yl)aristolactam I (dG-AAI); A3, 7-(deoxyadenosin-*N*^6^-yl)aristolactam II (dA-AAII).

The metabolic activation of BaP is catalysed predominantly by cytochrome P450-dependent monooxygenases (CYPs), mainly CYP1A1 and CYP1B1, in combination with microsomal epoxide hydrolase (mEH), resulting in the highly reactive BaP-7,8-dihydrodiol-9,10-epoxide (BPDE) capable of forming covalent DNA adducts (Fig. 1A) (Arlt et al., 2008, Stiborova et al., 2014a, Stiborova et al., 2014b). The effect of BaP on cell viability was similar in ES cells and MEFs at concentrations up to 5 μM (Fig. 2A and B). With a loss of viable cells of around 50% at 10 μM after 48 h of exposure, ES cells were more sensitive than MEFs. ES cells and MEFs were both capable of generating BaP-induced DNA adducts (Fig. 3A and B). The major DNA adduct (assigned spot B1) was previously identified as 10-(deoxyguanosin-*N*^2^-yl)-7,8,9-trihydroxy-7,8,9,10-tetrahydrobenzo[*a*]pyrene (dG-*N*^2^-BPDE) (Arlt et al., 2008). Interestingly, in ES cells we identified another adduct (assigned spot B2) that was more hydrophobic on PEI-cellulose than dG-*N*^2^-BPDE. In accordance with a recent study (Stiborova et al., 2014b) we suggest that adduct spot B2 is a guanine adduct derived from reaction with 9-hydroxy-BaP-4,5-epoxide. Using CYP1A1 reconstituted systems it was recently shown that the formation of dG-*N*^2^-BPDE (adduct B1) depended on the presence of epoxide hydrolase while adduct B2 was solely formed when CYP1A1 and NADPH:cytochrome P450 oxidoreductase (POR) only were present (Stiborova et al., 2014b). In MEFs two additional BaP-derived DNA adduct spots were detectable that were not structurally identified. No such adduct spots were detected in control (untreated) cells (data not shown). In ES cells BaP induced up to 126 ± 31 adducts per 10^8^ nucleotides at 10 μM after 48 h, with adduct levels being ∼3-fold lower after 24 h (Fig. 3A). BaP–DNA adduct levels in MEFs were manifoldly higher (Fig. 3B). The highest DNA adduct level in MEFs was observed at 2 μM after 48 h of BaP exposure (4583 ± 392 adducts per 10^8^ nucleotides), which was 44 times higher than in ES cells under the same experimental conditions. In a recent study using primary HUFs treated with 1 μM BaP for 48 h, levels of 175 ± 62 adducts per 10^8^ nucleotides were detected (Kucab et al., 2012), indicating that the response of MEFs to BaP can differ. However, it may also be difficult to try to directly compare these findings as strain differences (C57Bl/6 *versus* 129/Sv) and the p53 phenotype (*Hupki versus Trp53*) might have influenced the results between studies.

**Fig. 4:**
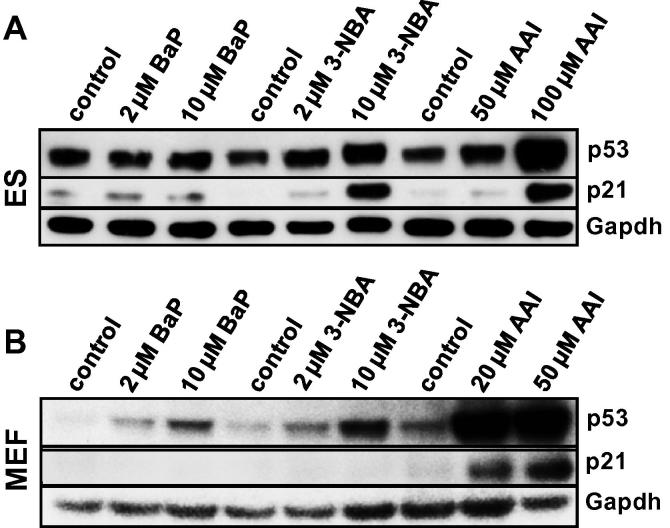
Western blot analysis of p53 and p21 (CDKN1A) protein expression in ES cells (A) and MEFs (B) derived mice on a C57Bl/6 genetic background carrying wild-type *Trp53* after exposure to BaP, 3-NBA and AAI for 48 h. Representative images of the Western blotting are shown and duplicate analysis was performed from independent experiments.

Because cellular levels of p53 protein increase via post-transcriptional mechanisms upon genotoxic stress (Hockley et al., 2008), we measured protein expression of p53 and its downstream target p21 (Fig. 4). p53 and p21 expression was not altered in ES cells after BaP exposure (Fig. 4A), however, a clear increase in p53 expression was observed in BaP-treated MEFs while p21 remained unchanged (Fig. 4B). These results were in line with the results obtained by ^32^P-postlabelling analysis. ES cells have been shown to contain a higher amount of p53 than differentiated cells (Solozobova and Blattner, 2010) and regulation of p53 is known to differ in ES cells and differentiated cells, thus the p53 response to DNA damage in these cell types may also be different (Liu et al., 2014, Solozobova et al., 2009).

**Fig. 5:**
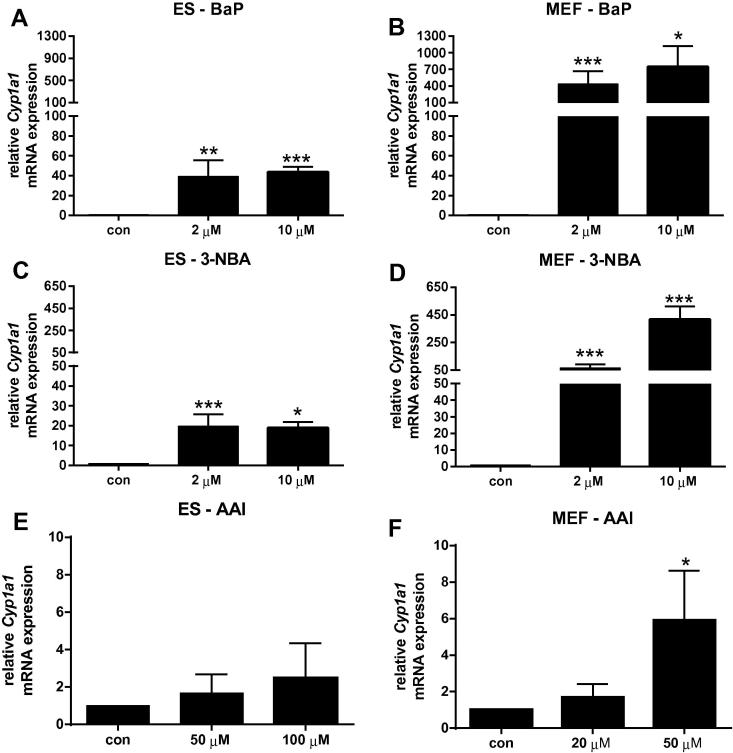
Gene expression of *Cyp1a1* in ES cells (*left panel*) and MEFs (*right panel*) derived from mice on a C57Bl/6 genetic background carrying wild-type *Trp53* after exposure to BaP (A and B), 3-NBA (C and D) and AAI (E and F) for 24 h. Values are the mean ± SD of three incubations; each sample was determined by three separate analyses. Basal *C_t_* values for *Cyp1a1* mRNA were 28.6 ± 0.5 and 36.5 ± 1.2 for untreated ES cells and MEFs, respectively. For statistical analysis the relative mRNA expression data was log2 transformed and analysed using a single sample *t-*test with Bonferroni correction against the population control mean of 0 (^∗^*p* < 0.05; ^∗∗^*p* < 0.01, ^∗∗∗^*p* < 0.005, different from control).

**Fig. 6:**
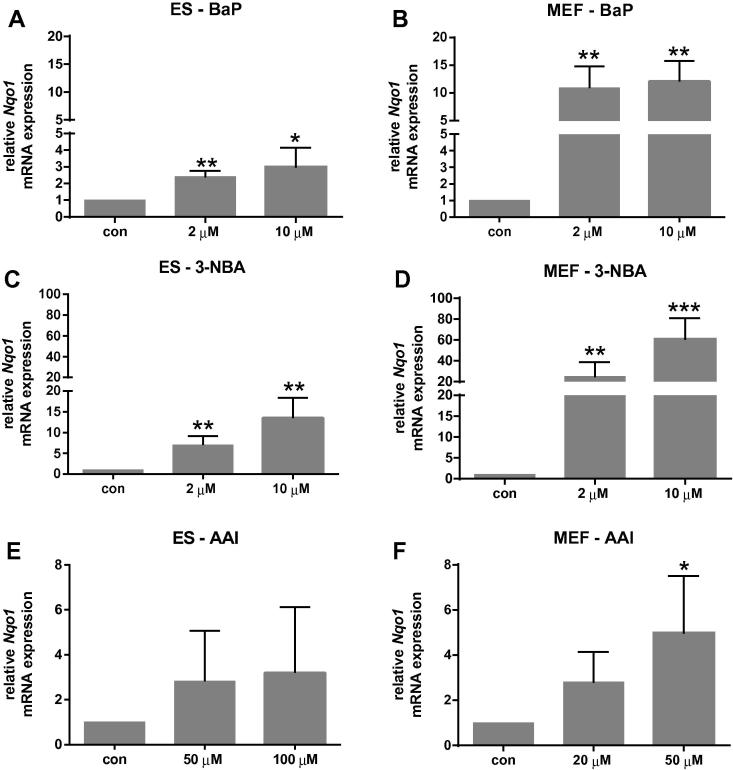
Gene expression of *Nqo1* in ES cells (*left panel*) and MEFs (*right panel*) derived from mice on a C57Bl/6 genetic background carrying wild-type *Trp53* after exposure to BaP (A and B), 3-NBA (C and D) and AAI (E and F) for 24 h. Values are the mean ± SD of three incubations; each sample was determined by three separate analyses. Basal *C_t_* values for *Nqo1* mRNA were 24.3 ± 0.3 and 25.7 ± 1.2 for untreated ES cells and MEFs, respectively. For statistical analysis the relative mRNA expression data was log2 transformed and analysed using a single sample *t-*test with Bonferroni correction against the population control mean of 0 (^∗^*p* < 0.05; ^∗∗^*p* < 0.01, ^∗∗∗^*p* < 0.005, different from control).

In order to determine whether the differences in BaP-induced DNA adduct levels observed between ES cells and MEFs could be due to differences in their metabolic competence, the expression of XMEs involved in BaP metabolism was evaluated. We therefore analysed *Cyp1a1* and *Nqo1* mRNA expression by RT-PCR. In BaP-treated ES cells expression of *Cyp1a1* was up-regulated ∼40-fold (Fig. 5A) independent of the BaP concentration used, which was in line with the observed BaP-induced DNA adduct levels. In MEFs BaP exposure resulted in a massive induction of *Cyp1a1* expression (Fig. 5B) and in comparison to ES cells this induction was ∼20-fold higher. Thus, these results suggest that MEFs have more BaP metabolising potential than ES cells and that the level of *Cyp1a1* expression can help to explain the differences in BaP–DNA adduct formation between both cell types. However, the lack of a suitable/sensitive antibody did not allow us to verify these results at the protein level of Cyp1a1 and it may be important to point out that gene expression does not always correlate with protein expression. *Nqo1* mRNA expression was induced after BaP exposure both in ES cells and MEFs (Fig. 6A and B), which is in line with previous studies using other mammalian cells (Hockley et al., 2006, Hockley et al., 2008). It is noteworthy that in the ToxTracker assay BaP required the addition of an exogenous metabolic activation system (*i.e.* liver S9 mix) to induce reporter activation in mouse ES Bscl2-tagged reporter cells (Hendriks et al., 2012), suggesting there are differences in the metabolic competence of ES cells of different origin.

### 3.2 Metabolic activation and DNA damage induced by 3-NBA in ES cells and MEFs

Bioactivation of 3-NBA is catalysed by nitroreductases such as NQO1 leading to *N*-hydroxy-3-aminobenzanthrone (*N*-OH-3-ABA) (Arlt et al., 2005, Stiborova et al., 2010). Further activation of *N*-OH-3ABA by *N*-acetyltransferases and/or sulfotransferases leads to the formation of reactive *N*-acetoxy and/or sulfooxy ester capable of forming DNA adducts (Fig. 1B) (Arlt et al., 2002). While BaP had only a small effect on cell viability in ES cells, 3-NBA was highly toxic to these cells; viability was already by ∼50% at 2 μM of 3-NBA (Fig. 2C). In comparison, 3-NBA treatment had little effect on cell viability in MEFs (Fig. 2D). The DNA adduct pattern induced by 3-NBA in ES cells and MEFs was the same, consisting of 4 major adducts (Fig. 3C and D). Three of these adducts were previously identified as 2′(2′-deoxyadenosine-*N*^6^-yl)-3-aminobenzanthrone (dA-*N*^6^-3-ABA; spot N1), *N*-(2′-deoxyguanosine-*N*^2^-yl)-3-aminobenzanthrone (dG-*N*^2^-3-ABA; spot N3), and *N*-(2′-deoxyguanosin-8-yl)-3-aminobenzanthrone (dG-C8-*N*-3-ABA; spot N4) (Arlt et al., 2006, Gamboa da Costa et al., 2009). DNA adduct formation by 3-NBA was time- and concentration dependent (Fig. 3C and D). In MEFs 3-NBA-induced DNA adduct formation was higher after 48 h, while adduct levels in ES cells were lower after 48 h. It is possible that DNA adduct formation in ES cells might have been compromised by the high level of cytotoxicity at 48 h. Using Western blot analysis we observed an increase in p53 protein expression in both cell types, but the downstream target p21 was only strongly induced in 3-NBA-treated ES cells (Fig. 4A and B). A strong p53 response has also been observed in other mammalian cells after 3-NBA treatment (Landvik et al., 2010). Further, it has been shown previously that 3-NBA induces a DNA damage response characterised by phosphorylation of ATM, Chk2/Chk1 and p53 (Oya et al., 2011), suggesting that 3-NBA-induced cell death, as seen in the ES cells (compare Fig. 2C), is a result of p53 activation.

The highest DNA binding by 3-NBA in ES cells was observed at 10 μM after 24 h with 863 ± 74 adducts per 10^8^ nucleotides (Fig. 3C). Interestingly, and in contrast to BaP, adduct levels for 3-NBA in MEFs were only 1.5-fold higher (1266 ± 188 adduct per 10^8^ nucleotides) under the same experimental conditions (Fig. 3D). DNA binding was highest in MEFs at 10 μM after 48 h with 2478 ± 455 adducts per 10^8^ nucleotides. Previously, in primary HUFs previously treated with 10 μM 3-NBA for 48 h, adduct levels were 680 ± 147 adducts per 10^8^ nucleotides (Kucab et al., 2012). As 3-NBA is predominantly activated by NQO1 (Arlt et al., 2005), the expression of *Nqo1* was studied in ES cells and MEFs by RT-PCR and revealed that *Nqo1* mRNA expression increased in both cell types up to ∼60-fold; the induction was higher in MEFs than in ES cells (Fig. 6C and D). This is in line with a previous study showing that Nqo1 protein levels were inducible in primary and immortal HUFs upon treatment with nitro-PAHs such as 1,8-dinitropyrene and 3-NBA (Kucab et al., 2012). However, that study also showed that there was not a clear relationship between nitro-PAH-induced DNA adduct formation and the expression of Nqo1, suggesting that other cytosolic nitroreductases such as xanthine oxidase might also contribute to the activation of nitro-PAHs like 3-NBA in HUFs (Kucab et al., 2012). As shown in Fig. 5C and D, 3-NBA also induced *Cyp1a1* mRNA expression, the induction in MEFs being manifoldly higher than in ES cells. Other studies have demonstrated the induction of Cyp1a1 protein levels in mouse Hepa1c1c7 cells after exposure to 3-NBA treatment (Landvik et al., 2010) and *in vivo* in rats treated with 3-NBA (Mizerovska et al., 2011, Stiborova et al., 2006, Stiborova et al., 2008).

### 3.3 Metabolic activation and DNA damage induced by AAI in ES cells and MEFs

The major activation pathway of AAI is nitroreduction, cytosolic NQO1 being the most efficient activating enzyme while CYP1A-mediated demethylation contributes to AAI detoxification (Fig. 1C) (Stiborova et al., 2014a, Stiborova et al., 2013). Exposure to AAI resulted in loss of cell viability of both ES cells and MEFs (Fig. 2E and F). However, in contrast to 3-NBA which showed strong cytotoxicity in ES cells, AAI cytotoxicity was higher in MEFs. We therefore chose 20 μM and 50 μM AAI in MEFs while ES cells were treated with up to 100 μM for DNA adduct analysis by ^32^P-postlabelling (Fig. 3E and F). The AAI-induced adduct patterns in ES cells and MEFs were the same and identical to the patterns observed in kidney and ureter tissue of AAN patients (Gokmen et al., 2013, Nortier et al., 2000). These adducts have previously been identified as 7-(deoxyadenosine-*N*^6^-yl)aristololactam I (dA-AAI; spot A1), 7-(deoxyguanosin-*N*^2^-yl)aristolactam I (dG-AAI; spot A2) and 7-(deoxyadenosin-*N*^6^-yl)aristolactam II (dA-AAII; spot A3) (Bieler et al., 1997, Schmeiser et al., 2014). DNA adduct formation by AAI was time- and concentration-dependent in ES cells with adduct levels being highest at 100 μM after 48 h (54 ± 27 adducts per 10^8^ nucleotides). In MEFs adduct formation increased with time at 20 μM but at 50 μM after 48 h resulted in lower adduct levels (compare Fig. 2F). As indicated above, it may be possible that the increased cytotoxicity at this condition may have impacted metabolic activation of the compound and/or DNA adduct formation. Highest DNA binding in MEFs was observed at 50 μM after 24 h with 2810 ± 1048 adducts per 10^8^ nucleotides which was 468-fold higher than the adduct levels observed under the same experimental conditions in ES cells (6 ± 3 adducts per 10^8^ nucleotides). AAI-induced DNA damage in MEFs was associated with a strong induction of the DNA damage response proteins p53 and p21 (Fig. 4B). Interestingly, AAI exposure also led to a strong p53 induction in ES cells and also subsequently its downstream target p21 but at considerably lower DNA adduct levels than in MEFs.

In ES cells neither *Nqo1* nor *Cyp1a1* mRNA expression was significantly altered after AAI treatment (Figs. 5E and 6E). In contrast, we found a significant induction of *Nqo1* and *Cyp1a1* in MEFs (Figs. 5F and 6F) but the levels of transcriptional alterations in MEFs are very small, and thus do not explain the differences of AAI–DNA adduct formation observed in the two cell types. Further, as the basal *Cyp1a1* and *Nqo1* mRNA expression levels in untreated ES cells and MEFs were only marginally different, if at all (see legends to Fig. 5, Fig. 6), this also did not provide an explanation for the huge differences in AAI–DNA adduct formation between cell types. Therefore we investigated whether the observed alterations in AAI-induced DNA damage are linked to epigenetic changes.

### 3.4 Potential impact of global DNA methylation on DNA damage induced by AAI in ES cells and MEFs

Tumours are characterized by a global reduction in DNA methylation (hypomethylation) and/or a locus-specific increase in DNA methylation (hypermethylation) (Esteller, 2008). DNA methylation can regulate gene expression and it has been shown in cancer cells that DNA hypermethylation of CpG islands near tumour suppressor genes switches off the expression of these genes (Tommasi et al., 2014). Further, it has been suggested that epigenetic mechanisms may function as an interface between environmental factors and the genome and that aberrant epigenetic changes associated with environmental exposures might deregulate not only key cellular processes such as DNA damage response and DNA repair but also carcinogen metabolism (Herceg and Vaissiere, 2011). Several environmental pollutants have been shown to affect DNA methylation in mammalian cells *in vitro*. Tabish et al. (2012) demonstrated for example that benzene, hydroquinone, styrene, carbon tetrachloride and trichloroethylene induced global DNA hypomethylation in human TK6 cells. However, little is known about equivalent mechanisms in embryonic stem cells or MEFs.

**Fig. 7:**
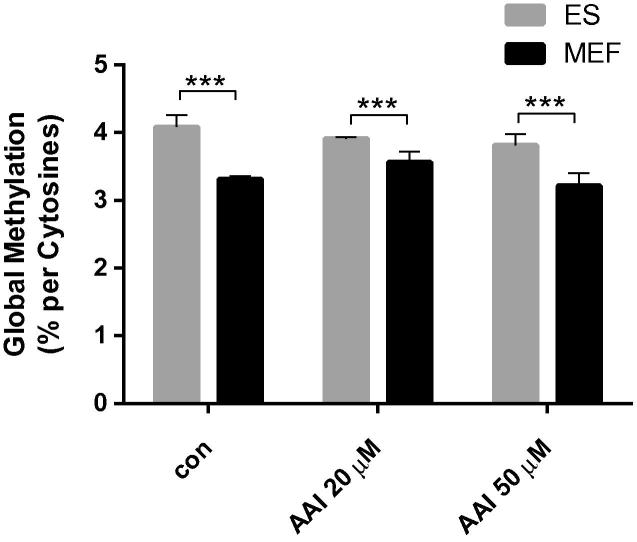
Global methylation in ES cells and MEFs derived from mice on a C57Bl/6 genetic background carrying wild-type *Trp53* and the effect of AAI exposure on global methylation. Values are the mean ± SD of at least three incubations. Statistical analysis was performed by one-way ANOVA followed by Tukey post-hoc test (^∗∗∗^*p* < 0.005, different from ES cells).

We assessed global DNA methylation in ES cells and MEFs derived from the PLF mouse after AAI exposure using capillary electrophoresis with laser induced fluorescence (Krais et al., 2011). It has been reported that global DNA methylation decreases as embryonic stem cells undergo differentiation (Smith and Meissner, 2013). Indeed, we found that global DNA methylation of the ES cells was 4.08 ± 0.05% 5-methylcytosine while in MEFs it was 3.31 ± 0.18% 5-methylcytosine (Fig. 7). However, AAI treatment did not alter global methylation. Nevertheless, covalent modification of DNA and histone proteins, the core components of chromatin, provide a mechanisms for heritable regulating gene expression by changing the accessibility of DNA to interacting proteins (Jin et al., 2011). We thus hypothesize that the higher methylation levels in ES cells might lead to a better protection of the genome due to higher chromatin density and lesser accessibility of the DNA. However, differences in DNA damage between ES and MEF cells could be due to other underlying mechanisms, such as DNA repair and/or apoptosis (Roos et al., 2007, Tichy and Stambrook, 2008).

## 4 Conclusions

In this study we showed that ES cells and MEFs derived from mice on a C57Bl/6 genetic background carrying wild-type *Trp53* have the metabolic competence to activate a number of environmental carcinogens. Our results clearly indicate that MEFs not only have a higher metabolic capacity than ES cells but also that the metabolic capacity depends on the carcinogen studied. Thus, the generation of sets of ES cells and MEFs derived from the PLF mouse (on the same genetic background) harbouring point mutations in *TP53* will allow comparative functional analyses of p53 in cells with a matched genetic background. Recently PLF-derived MEFs carrying common tumour mutants R248W and R273C were compared with MEFs carrying *TP53* mutants associated with AA exposure, namely N131Y, R249W and Q104L (Odell et al., 2013). Based on a number of biological endpoints tested including cell proliferation, migration, growth in soft agar, apoptosis, senescence and gene expression it was demonstrated that the N131Y mutant had a phenotype more related to the common tumour mutants R248W and R273C, whereas behaviour of clone Q104L resembled more the phenotype of a cell with wild-type p53 (Odell et al., 2013). Taken together, these and our studies show that the cellular behaviour of these novel mutants can be studied after carcinogen exposure but that carcinogen treatment conditions must be optimised prior to initiating any assay to study p53 function and that carcinogen metabolism depends on the cell type studied.

## Conflict of Interest

The authors declare that there are no conflicts of interest.

## Transparency Document

Transparency document
